# Chloroplasts and Plant Immunity: Where Are the Fungal Effectors?

**DOI:** 10.3390/pathogens9010019

**Published:** 2019-12-24

**Authors:** Matthias Kretschmer, Djihane Damoo, Armin Djamei, James Kronstad

**Affiliations:** 1 Michael Smith Laboratories, Department of Microbiology and Immunology, University of British Columbia, Vancouver, BC V6T 1Z4, Canada; kretschm@msl.ubc.ca (M.K.); djihane.damoo@msl.ubc.ca (D.D.); 2 Leibniz Institute of Plant Genetics and Crop Plant Research (IPK) OT Gatersleben Corrensstrasse 3, D-06466 Stadt Seeland, Germany; djamei@ipk-gatersleben.de

**Keywords:** fungal phytopathogenesis, reactive oxygen species, light-harvesting complex, effector

## Abstract

Chloroplasts play a central role in plant immunity through the synthesis of secondary metabolites and defense compounds, as well as phytohormones, such as jasmonic acid and salicylic acid. Additionally, chloroplast metabolism results in the production of reactive oxygen species and nitric oxide as defense molecules. The impact of viral and bacterial infections on plastids and chloroplasts has been well documented. In particular, bacterial pathogens are known to introduce effectors specifically into chloroplasts, and many viral proteins interact with chloroplast proteins to influence viral replication and movement, and plant defense. By contrast, clear examples are just now emerging for chloroplast-targeted effectors from fungal and oomycete pathogens. In this review, we first present a brief overview of chloroplast contributions to plant defense and then discuss examples of connections between fungal interactions with plants and chloroplast function. We then briefly consider well-characterized bacterial effectors that target chloroplasts as a prelude to discussing the evidence for fungal effectors that impact chloroplast activities.

## 1. Introduction

Plastids are dynamic plant organelles that differentiate during development into photosynthetic chloroplasts as well as leucoplasts, chromoplasts, and additional subtypes [1]. In the context of plant immunity, most studies focus on chloroplasts, which are responsible for the formation of energy equivalents, such as ATP and NADPH during photosynthesis. These energy equivalents can be used during and after carbon fixation to produce primary carbon-containing metabolites, such as sugars, starch, nucleotides, amino acids and fatty acids/lipids. Plastids, and more specifically chloroplasts, also make important contributions to plant defense against pathogens, including participation in the pathogen-associated molecular pattern (PAMP), triggered immunity (PTI) and effector-triggered immunity (ETI) [2,3,4,5,6,7,8]. As a basal layer of plant defense, PTI is usually triggered by conserved PAMPs on pathogens (e.g., bacterial flagellin and fungal chitin) or by damage-associated molecular patterns (DAMPs, such as oligogalacturonides or cellobiose) that may originate from the plant cell wall [2,4,5]. ETI is a second layer of plant immunity that requires the recognition of effectors by resistance (R) proteins leading to a hypersensitive response (HR) [2,4,5]. Key reactions during defense include the synthesis of secondary metabolites and phytoalexins, the formation of reactive oxygen species (ROS) and nitric oxide (NO), calcium oscillations, stomatal closure, apoplastic alkalization, cell wall strengthening and the expression of plant defense proteins including pathogenesis-related proteins (PRs) [4,5,9,10]. Chloroplasts are the sites of production of ROS, calcium oscillations, and the synthesis of plant defense molecules, including jasmonic acid (JA) and salicylic acid (SA), that are critical components of the plant defense strategy against necrotrophic (JA) and biotrophic pathogens (SA). Overall, it is clear that chloroplasts are a critical hub connecting plant defense responses to primary anabolic functions. 

In this review, we first briefly summarize the contributions of the chloroplast to immunity to set the stage for understanding potential targets of pathogen attack on the plastid. Our focus here is primarily on fungal pathogens, and readers are directed to several excellent recent reviews on the role of the chloroplast in interactions with viral and bacterial pathogens [4,5,11]. We initially discuss recent examples of chloroplast functions that are impacted by interactions with fungi and specifically consider chloroplast contributions to defense against fungi. Information on bacterial interactions is included to reinforce findings of key chloroplast functions that mediate defense or serve as targets of pathogen attack. We then use the emerging information about bacterial effectors that target chloroplast functions as a guide to consider recent studies on fungal effectors. Effectors have also been studied in detail in oomycetes, and we refer readers to recent papers and reviews for coverage of these pathogens [12,13,14]. Finally, we discuss future directions, including the prediction that fungal effectors targeting the chloroplast will be prominent contributors to disease.

## 2. An Overview of Chloroplast Contributions to Plant Immunity 

The phytohormone JA is synthesized in the chloroplast and plays a crucial role in plant immunity [4,5]. In particular, JA (along with ethylene (ET)) signaling contributes to defense against necrotrophic pathogens but is considered to be ineffective against biotrophic pathogens [5]. JA is derived from linolenic acid that is released from chloroplast membranes and oxidized to form 12-oxo-phytodienoic acid (OPDA) by the action of allene oxide synthases and cyclases. OPDA is then exported from the chloroplast to form JA in the peroxisome [4,5,8]. Exogenous JA is known to reduce the chlorophyll content of potato leaves, to reduce electron transport of the photosystems (PSI and PSII), and to impact carbon fixation by downregulation of chlorophyll biosynthesis and photosynthesis-related genes [5]. The phytohormone ET also regulates chlorophyll content, PSI and PSII efficiency, and rubisco activity in *Arabidopsis* and tobacco in an age-dependent manner [5]. 

As a defense hormone, SA is antagonistic to ET and JA in its activity to induce effective plant defense mainly against hemibiotrophic and biotrophic pathogens. Two SA biosynthesis pathways that involve chloroplast functions are proposed in plants. One pathway converts chorismate to isochorismate via isochorismate synthase (ICS1) in the chloroplast with subsequent conversion of isochorismate to SA in the cytosol [15]. A second pathway accounts for ~10% of SA synthesis by converting isochorismate to SA via the phenylalanine ammonia-lyase pathway in the cytosol [3,4,8,15,16]. Phenylalanine is also synthesized in the chloroplast from chorismate [5]. Thus, in both cases, chloroplasts play a major role in SA biosynthesis. SA stimulates photosynthesis at low externally applied concentrations (10^−5^ µM) but is inhibitory at higher concentrations. Although not the mobile signals, SA or derivatives, such as methyl-SA, are also involved in systemic acquired resistance after a local primary infection leading to HR [4,5]. 

The production of ROS is also a key defense contribution of chloroplast metabolism. The photosynthetic electron transport chains of PSI and II of the chloroplast are major players in providing electrons for free radical formation (e.g., singlet ^1^O_2_ and superoxide O_2_^−^). PSI produces O_2_^−^ that is converted to H_2_O_2_ by superoxide dismutases, while PSII mainly forms singlet oxygen ^1^O_2_ [3]. ROS have several plant defense activities besides the direct killing of pathogen cells. These activities include involvement in both PTI and ETI with contributions to cell wall strengthening, signaling and HR induction. For PTI, PAMPs are known to trigger the downregulation of PsbS, a thylakoid sensor that regulates non-photochemical quenching (NPQ); this regulation drives the electrons from the photosystems to form increased amounts of oxygen radicals rather than dissipation of the free energy as heat [4,17]. The second extended high amplitude ROS burst is important for plant defense responses in the context of ETI and HR. For example, tobacco with reduced chloroplastic ROS formation showed reduced HR when challenged with *Xanthomonas campestris* pv. *vesicatoria* [5]. The activation of the mitogen-activated protein kinases MPK3 and MPK6 is important for ETI and leads to repression of photosynthesis-related genes and induces plant defense/secondary metabolism genes [10]. This uncoupling of photosynthesis by inhibiting PSII leads to the overpowering of the NPQ and the rapid accumulation of ROS under light conditions [10]. MPK3/6 deletion mutants are compromised in HR, and the expression of a chloroplast-targeted flavodoxin reduced inhibition of photosynthesis, ROS formation and HR [10]. Thus, ETI depends on the MPK3/6 signaling cascade during light conditions for ROS formation, which might also explain why plants are more resistant during daylight compared to dark phases where no ROS is formed. This information also indicates that the global reprogramming of chloroplast functions, such as the downregulation of photosynthesis versus the upregulation of plant defense/secondary metabolism, is an active plant defense strategy, rather than a pathogen-induced phenotype [9,10]. However, plant pathogenic microbes are known to secrete effectors that target several chloroplastic functions, as discussed further below.

Chloroplast-to-nucleus retrograde signaling is crucial for the proper functioning and assembly of the photosynthetic apparatus [18]. Similarly, changes in the developmental or metabolic states of the chloroplast result in severe changes in the transcript profiles of nuclear genes [19]. This suggests that the chloroplast may act as an environmental sensor, mediating environmental stresses to regulate the transcription of certain nuclear genes. In the context of disease, lesion mimic mutants in plants form spontaneous lesions that resemble HR, and some of these mutations are in nuclear genes that encode chloroplast proteins [20]. Retrograde signaling is thought to involve Ca^2+^ sensing and a ROS signal that could be transferred to the nucleus via stromule bridges originating from the chloroplasts and observed during pathogen attacks [4,5,6,8,10,17,21,22,23,24,25]. Stromules are stroma-filled, tube-like extrusions originating from the chloroplast [21,22,23,24,25]. They depend on the chloroplast unusual positioning 1 protein (CHUP1) that promotes stromule formation along microtubule-guided extensions, with actin microfilaments providing anchoring points [21,22,23,24,25]. Ca^2+^ fluxes are induced during pathogen recognition and include Ca^2+^ spikes in the chloroplast after PTI is triggered by flagellin or chitin. Stromal Ca^2+^ spikes are regulated by a thylakoid membrane-bound Ca^2+^ sensing protein [4,6,17]. Ca^2+^ sensing in concert with ROS was shown to be responsible for PTI and ETI by regulating Ca^2+^ fluxes, SA biosynthesis and the induction of nuclear-encoded defense genes [6]. For example, PTI is compromised in a mutant defective in Ca^2+^ sensing with reduced stomatal closure, reduced callose deposition and phytoalexin formation after the plant was challenged with *Pseudomonas syringae*. ETI, in this situation, was severely delayed [5]. 

Retrograde signaling during plant defense further involves the chloroplast protein N-receptor interacting protein 1 (NRIP1) and the metabolites 3-phosphoadenosine-5-phosphate (PAP) and C-methyl-D-erythritol-2,4-cyclopyrophosphate (MEcPP) [3,4,8,25,26]. NRIP1 is transferred from the chloroplast to the nucleus during plant defense activation by the N resistance protein (against the tobacco mosaic virus), which is thought to involve stromules [8,21]. PAP originates in the cytosol but is degraded in the chloroplast by the phosphatase SAL1. The SAL1-PAP pathway, in turn, was shown to regulate the SA and JA biosynthesis pathways [26]. For example, *sal1* mutants displayed downregulation of SA and JA signaling pathways and showed enhanced symptom formation upon infection with both hemibiotrophic and necrotrophic bacterial pathogens [26]. MEcPP is an intermediate of the MEP pathway, which is a precursor for isoprenoids and the hormones GA and ABA. Under stress situations, MEcPP accumulates and promotes the expression of ICS1, leading to higher SA levels and increased SA defense signaling [4]. PAP and MEcPP are redox-regulated, and thus light-dependent ROS formation may potentiate these signals [4].

In addition to PAP and MEcPP retrograde signaling, targets of pathogen interference may involve the genome uncoupled (GUN) genes characterized in *Arabidopsis thaliana*. An unbiased forward genetic screen led to the identification of six GUN genes [27,28]. GUN1 is a chloroplast-localized protein, and a *gun1* mutant shows increased susceptibility to photooxidative stress [29,30]. Similar phenotypes can be observed in plants treated with inhibitors that target de novo protein synthesis in plastids or carotenoid biosynthesis (e.g., lincomycin and norflurazon, respectively) [28]. In addition, a co-IP experiment revealed that GUN1 interacts with many proteins involved in the maintenance of the chloroplast proteome as well as chaperones, suggesting it plays a role in coordinating chloroplast protein import and protein degradation [31]. The other GUN proteins encode enzymes involved in tetrapyrrole metabolism, and GUN2, GUN3 and GUN6 are involved in heme metabolism leading to the suggestion that heme may be a retrograde signal [18].

It is clear that retrograde signaling plays a major role in chloroplast biogenesis and plant immunity. However, it is unknown if several individual signals lead to retrograde signaling or if multiple signals are connected in a network required to control the expression of nuclear-encoded chloroplast proteins [18]. Pathogen effectors that directly target the retrograde signaling pathway in plants have not been identified so far. 

## 3. Impact of Fungi on Chloroplast Functions

### 3.1. Chloroplast Morphology and Position

Changes in chloroplast morphology and position have been observed during disease. For example, the infection of rice by the fungal pathogen *Rhizoctonia solani* severely disturbs chloroplast morphology and function [32]. Examination of diseased tissue revealed structural disintegration of chloroplast membrane structures (grana-, thylakoid and stroma organization) at 3 days post-infection. It was further shown that the chloroplasts at this time were the main source for ROS formation, with accompanying reduced photosynthetic performance. Factors such as maximum quantum yield of PSII, electron transport rate and non-photochemical quenching were markedly reduced [32]. In contrast, genes encoding secondary metabolism and plant defense functions associated with chloroplasts showed increased expression. Consequently, metabolites, such as sinapic acid (phenylpropanoid pathway) and alpha-linolenic acid (JA biosynthesis pathway), accumulated in higher amounts [32]. 

The activation of plant defense often results in chloroplast repositioning from an optimal position for photosynthesis to locations in proximity to the invading pathogen or the nucleus. For example, chloroplasts in *Nicotiana benthamiana* tend to accumulate close to the nucleus with consequent stromule formation during PTI and ETI defense responses, during bacterial or viral infection, or during the transient expression of viral proteins, such as REP or p50 [33]. Exogenous H_2_O_2_ is able to trigger chloroplast movement in tobacco, and chloroplast aggregation around nuclei is reduced upon inhibition of ROS formation by NADPH-oxidase inhibitors or external application of ROS scavengers, such as Tiron or dimethylthiourea [33].

### 3.2. Chloroplasts and ROS Generation

ROS generated in chloroplasts is a major contributor to plant immunity. For example, the expression of a cyanobacterial chloroplast-targeted flavodoxin (which reduces ROS accumulation) in transgenic tobacco reduced the symptoms of infection by the necrotrophic fungus *Botrytis cinerea* or the hemibiotrophic bacterium *X. campestris* [34,35,36]. The transgenic plants infected with *B. cinerea* showed less tissue damage and reduced fungal growth as well as changes in photosynthetic activity and plant defense responses [34]. In particular, mycelial growth was reduced by 67–90% in the transgenic plants expressing the cyanobacterial flavodoxin protein compared to wild-type plants during a time course of infection [34]. Several photosynthetic parameters, such as maximum quantum yield, photosynthetic performance index and electron transfer efficiency, were inhibited to a lesser degree in plants expressing flavodoxin. Furthermore, wild-type plants showed fewer intact active reaction centers of the PSII per leaf area compared to the flavodoxin-expressing lines [34]. Phytoalexin accumulation was also reduced in the infected plants expressing flavodoxin, and the expression of plant defense genes, such as glucanases, chitinases or PR1, was delayed compared to wild-type plants. Overall, it appears that chloroplast production of ROS contributes to the virulence of *B. cinerea* and that decreased ROS accumulation in the transgenic flavodoxin plants provides some protection against a necrotrophic pathogen. This conclusion is supported by a recent study on the contribution of chloroplast-produced tocopherols as antioxidants during infection of *Arabidopsis* with *B. cinerea* [37]. In this case, mutants with T-DNA insertions in *VTE1* (encoding tocopherol cyclase) or *VTE4* (encoding γ-tocopherol methyltransferase) were used to alter tocopherol composition [37]. In particular, a *vte1* mutant lacking α and γ-tocopherols and a *vte4* mutant that accumulates γ-tocopherol but lacks α-tocopherol both showed delayed resistance to *B. cinerea*. Lipid peroxidation also increased during infection with *B. cinerea*, which correlated with increased fungal biomass and fungal virulence. The altered susceptibility in the *vte1* and *vte4* mutants may be due to delayed linolenic acid processing leading to reduced JA formation [37].

The contribution of chloroplastic ROS for defense against biotrophic pathogens is illustrated by a recent study with the Wheat Kinase START1 (*WKS1*) gene [38]. *WKS1* is a race nonspecific resistance gene that encodes a protein with a serine/threonine kinase domain and a steroidogenic acute regulatory protein-related lipid transfer (START) domain. Partial plant resistance against the rust fungus *Puccinia striiformis* f. sp. *tritici* is seen in wheat transformed with multiple copies of the *WKS1* gene originally identified in wild tetraploid wheat. The WKS1 protein is located in the chloroplast, and was shown to interact with and ultimately phosphorylate a thylakoid-associated ascorbate peroxidase, thus limiting ascorbate detoxification of ROS leading to a slow cell death response and partial plant resistance [38]. The impact of ROS in the defense against hemibiotrophs is illustrated by an analysis of *Arabidopsis* plants expressing a ferrodoxin that is a major distributor of electrons in plastids [39]. Deletion of the gene encoding the plastid-localized ferredoxin FD2 led to increased virulence of the bacterial pathogen *P. syringae,* and this was correlated with a reduction in ROS formation during PTI [39]. Plants lacking FD2 showed dramatically induced JA formation, while SA formation was unchanged compared to wild type plants. This hormone imbalance induced JA-dependent defense gene expression and further led to reduced expression of SA biosynthetic genes (e.g., *ICS1*) and SA-dependent genes (e.g., *PR1*). In ETI with *P. syringae* containing *avrRpt2*, plants lacking flavodoxin produced ~2 times more H_2_O_2_ and consequently were more resistant than wild-type plants [39]. Ferredoxin2 was found to be located in stromules, which indicates a potential contribution to retrograde signaling [39]. 

Given that ROS functions play an important role during plant immunity, it is not surprising that the light-harvesting complexes, especially PSII, appear to be an integral part of plant defense. For example, the light-harvesting complex II protein LHCB5 is phosphorylated during infection of rice by the rice blast fungus *Magnaporthe oryzae* in a light-dependent manner [40]. Plant resistance to blast was increased in high light conditions for some rice lines, and this was correlated with expression changes in LHCB5 caused by promoter variations in the different rice lines [40]. Plants overexpressing LHCB5 were significantly more resistant to *M. oryzae*, while RNAi knockdown lines were hypersusceptible. The resistance was linked to differences in ROS formation with the overexpression line showing increased ROS amounts. LHCB5 phosphorylation was both dependent on *M. oryzae* infection and on light intensity, and specific phosphorylation at threonine 24 was responsible for the ROS burst and HR formation in a chloroplast-dependent manner [40]. In this context, it is also interesting that the phosphorylation state of the photosynthesis-related chaperonin-60 is important for plant immunity in *A. thaliana* during infection with the bacterium *X. campestris* pv. *campestris* [41]. Chaperonin-60 is the target of the chloroplast-localized protein phosphatases PP2C62/26, and the deletion of genes for these phosphatases increased plant resistance [41].

### 3.3. Chloroplast Pigments and Carbohydrate Metabolism

Several studies establish links between fungal disease and carotenoid or chlorophyll biosynthesis or catabolism. For example, virus-induced silencing of carotenoid or chlorophyll biosynthesis at the phytoene desaturase or Mg-chetalase H steps, respectively, led to a faster and stronger appearance of HR symptoms (likely due to higher ROS accumulation) during an infection of wheat with the hemibiotrophic fungus *Zymoseptoria tritici* [42]. The fungus *Z. tritici* switches to a necrotrophic attack at later stages of infection, and sporulation of the pathogen was impaired in plants with reduced chlorophyll biosynthesis [42]. This result indicates that modification of pigment biosynthesis can make a strong contribution to plant resilience to pathogen attack. Interestingly, earlier studies demonstrated that loss of the chloroplast protein Les22, a uroporphyrinogen decarboxylase involved in chlorophyll biosynthesis, results in spontaneous lesions that resemble an HR [43]. This example with the Les22 protein is characteristic of the phenomenon observed in lesion mimic mutants of several plant species (e.g., rice, maize and the model plant *Arabidopsis*) in which spontaneous HR like symptoms, the hallmark of ETI, are observed without a pathogen being present [44]. Lesion mimic mutations have also been identified in the maize (*ZmLls1*) and wheat (*TaLls1*) genes encoding pheophorbide *a* oxygenase (PaO) for chlorophyll breakdown [45]. *TaLls1* expression is induced in wheat upon infection with *P. striiformis* f. sp*. tritici* or wounding, and silencing of the gene increased tolerance to *P. striiformis* [45].

The biotrophic smut fungus *Ustilago maydis* also appears to influence chloroplast function, and this fungus provokes the formation of white tumors surrounded by normal green tissues [46]. Tumors on maize incited by *U. maydis* are chlorotic with all major chloroplast pigments severely reduced, especially chlorophyll b (41%) [47]. Overall, the genes for chlorophyll biosynthesis, photosynthesis complex proteins and CO_2_ fixation functions showed severely reduced expression in tumors. On average, most of the genes related to photosynthesis showed a 3–10 fold reduction in expression in tumors compared to uninfected plants [46]. Other chloroplast functions, such as fatty acid and lipid biosynthesis, amino acid biosynthesis, and secondary metabolism functions of the shikimate pathway and the phenylalanine biosynthetic pathway, were also downregulated [47]. Impairment of chloroplast functions in a chloroplast biosynthesis mutant or by treatment of plants with herbicides (e.g., glyphosate^®^) resulted in increased disease symptom formation upon *U. maydis* inoculation. This result indicates that chloroplasts are integral for plant immunity in this pathosystem. In contrast, chloroplast-related carbohydrate functions, including genes for several starch biosynthesis proteins (e.g., *sbe1* and *ae1*), were upregulated 2.5 to 14 fold in tumors. Consequently, more starch was seen in tumor tissue compared to uninfected stems of maize seedlings. Interestingly, the *sugary1* maize line (with altered starch biosynthesis) was more resistant than the wild type line during seedling infection with *U. maydis* [47].

*Ustilago maydis* also exerts a metabolic priming influence on the maize defense response by secreting the enzyme chorismate mutase (encoded by the effector gene *cmu1*) into the cytosol of host cells. The enzyme acts to redirect the shikimate pathway and promote reduced SA production [48]. Recently, one of 20 maize-encoded kiwellins, *Zm*KWL1, was found to disarm the Cmu1 chorismate mutase by hindering the access of the substrate to the enzyme’s active site [49,50]. *Zm*KWL1 belongs to a subgroup of kiwellins found exclusively in cereal plants, and *Zm*KWL1 interacts exclusively with *U. maydis* Cmu1 in vitro and not with the maize version of chorismate mutase [49,51]. It was proposed that *Zm*KWL1 is secreted into the apoplast, where it binds Cmu1 and presumably decreases the import of the enzyme into host cells [49].

Variegated plants that show green and white sectors also provide an informative approach to examine the contributions of chloroplast functions to plant immunity. The photosynthetic-active green tissue shows normal chloroplast development and function, while the white sectors display a sink-like behavior with undeveloped, non-photosynthetically active plastids. The *immutans* mutation in *Arabidopsis* causes a variegated phenotype due to variations in the plastid terminal oxidase (PTOX) that disturb carotenoid and chlorophyll biosynthesis during chloroplast biogenesis [52]. Analysis of the mutant showed that white sectors experience ROS stress and demonstrated remodeling of the plant cell wall with reduced lignin amounts and cellulose microfibrils as well as changes in galactomannans and xyloglucans amounts/distribution [52]. Although tests with fungal pathogens have not been reported, white sectors challenged with the bacterial pathogen *P. syringae* showed reduced plant defense gene activation (e.g., PR1 and PR5 expression), reduced callose deposition and higher colonization of the tissue by the bacteria compared to green sectors or wt plants [52]. Perhaps the higher bacterial growth reflects greater nutrient availability due to the sink behavior of the white sectors.

In the context of the impact of fungi on chloroplast function, a long-standing observation is that certain biotrophs, such as rust or powdery mildew fungi, and some hemibiotrophic pathogens, cause so-called green islands of photosynthetically active tissue around infection sites [53]. These islands occur in a background of otherwise yellowing and senescent tissue. Photosynthetic activity is maintained in green island tissue, although it is generally reduced compared to healthy tissue. For example, overall net photosynthesis can be reduced by as much as 32%, and quantum yield can be 47% lower in the green islands on barley leaves infected with the powdery mildew pathogen *Blumeria graminis* [53,54]. Chloroplast organization and morphology appear to be normal between green islands or uninfected tissue, and photosynthetic capacity may be reduced by an initial loss of chlorophyll with subsequent re-biosynthesis of new chlorophyll (the re-greening hypothesis). It has also been proposed that the chlorophyll in green islands is retained in chloroplasts (the chlorophyll retention hypothesis) and that cytokinins, possibly produced by the biotrophic fungi, play an important role in green island formation [53]. In general, the process of green island formation requires considerable additional investigation to uncover the underlying molecular mechanisms.

### 3.4. Positive Influences of Fungi on Chloroplast Function

In general, the study of beneficial fungi and their interactions with chloroplasts may provide important insights for improving plant productivity and for understanding the impact of pathogenic fungi on chloroplasts. It is well established that endophytic and mycorrhizal fungi can increase plant performance and photosynthetic capacity. For example, the fungal endophyte *Epichloe typhina* increased the abundance of PSI proteins (PsaC, Lhca2) and PSII proteins (D1, Lhcb3) of its host plant orchard grass by 2–3 fold [55]. Chlorophyll accumulation increased by 33%, especially chlorophyll b, and overall net photosynthesis was increased by ~32% at saturated light conditions [55]. Similarly, the interaction of arbuscular mycorrhizal fungi with watermelon also triggers increased carotenoid and chlorophyll a and b amounts and improved photosynthesis parameters such as net photosynthesis rate, PSII maximum yield, actual photochemical quantum and photochemical quenching [56]. Abiotic salt stress negatively influences these photosynthetic parameters, but the negative effects can be alleviated by mycorrhizal colonization of the plant roots [56]. Interestingly, it was previously found that the plastid-localized proteins CASTOR and POLLUX are essential for symbiotic plant interactions in lotus with nitrogen-fixing bacteria or arbuscular mycorrhizal fungi [57]. Loss of either protein leads to the inhibition of cytoplasmic Ca^2+^ spiking and abortion at early infection stages. During the bacterial interaction, infection threads are not formed, while during the mycorrhizal interaction, the fungus is unable to establish root epidermal cell invasion. The arbuscular mycorrhizal interaction in alfalfa also significantly reduces the negative effects of the herbicide atrazine, a PSII electron transport inhibitor, on chloroplast structure and PSII performance, but does not mitigate the negative effects on chloroplast pigment accumulation [58]. In contrast, secondary metabolites, such as coriloxine or quinone derivatives from the endophytic fungus *Xylaria feejeensis,* have negative effects on ATP synthesis of spinach thylakoid preparations, and these compounds can further be chemically modified to show even greater effects against photosynthetic functions [59].

As mentioned above, plant growth and photosynthetic capacity are generally known to increase during interactions with beneficial microorganisms. Therefore, it seems counter-intuitive to observe positive effects on chloroplast functions during a necrotrophic fungal attack, although this phenomenon has been recently observed for the fungal pathogen *Alternaria alternata* [60]. Volatile compounds of beneficial microorganisms are known to have positive effects on plant growth and photosynthesis capacity, and the work with *A. alternata* indicates that volatiles from the necrotrophic fungus also have similar effects on plants [60]. The influences of the volatiles included increased photosynthesis parameters, such as PSII operating efficiency and photochemical quenching, and increased chlorophyll content in proximity to *A. alternata* volatiles [60]. These phenotypes depended on the redox state of the plant, and analysis of the thiol redox proteome indicated that volatile compounds lead to a reduced protein state, especially for photosynthesis-related proteins. These phenotypes are controlled by the chloroplast protein NADPH-dependent thioredoxin reductase [60]. The consequences of improved chloroplast functions for the necrotrophic plant pathogen require further investigation.

## 4. Effectors and Chloroplasts

**Figure 1:**
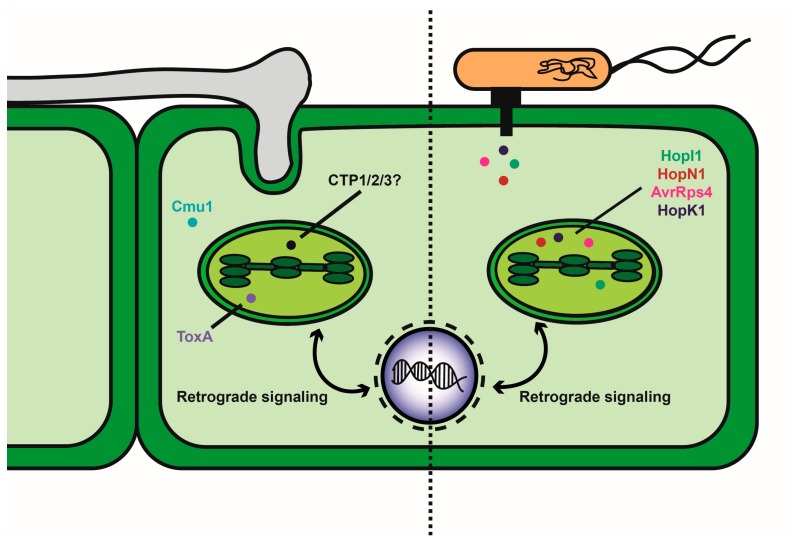
Diagram of fungal and bacterial delivery of known and candidate effectors that influence chloroplast function. The left side of the diagram depicts the interaction of a fungal pathogen with a plant cell to deliver effectors by mechanisms that could involve colonization of the apoplast, penetration of host cells or formation of haustorial feeding structures. Known effectors (Cmu1 and ToxA) are shown with ToxA localized to the chloroplast. Candidate effectors (CTP1, 2 and 3) are localized to the chloroplast, but their impact of function is not yet known. The right side of the diagram shows the delivery of effectors by bacterial pathogens via a type III secretion system. Representative effectors delivered by *Pseudomonas syringae* are listed.

Given that the chloroplast plays a key role in plant immunity, it is reasonable that the organelle would be a prime target of effector proteins introduced by pathogens [8]. Plant pathogens secrete a cocktail of effector proteins or virulence factors that are known to act in the apoplast or the cytoplasm, where they may target specific organelles [7,61,62,63,64,65,66] (Figure 1). While effector proteins are very diverse, with different mechanisms of action, ultimately, what they have in common is their ability to facilitate pathogen proliferation in the host in the absence of direct or indirect detection by corresponding compatible resistance proteins (R) to trigger ETI. In compatible interactions, effectors contribute to virulence by suppressing the plant immune response, by interfering with the host’s physiology to promote nutrient acquisition, by influencing organelle function and gene expression, or by as yet unknown mechanisms [64,66,67]. 

### 4.1. Examples of Effectors that Target the Chloroplast

Several bacterial pathogens secrete effectors that target chloroplast functions [7,68,69]. Much of what is known about these effectors comes from studies with *P. syringae* pathovars and, more specifically, the interaction of *P. syringae* pv. *tomato* (*Pst*) strain DC3000 with *A. thaliana* [70,71]. Several *P. syringae* effectors are known to manipulate chloroplast functions, including the well-characterized protein HopI1, an effector with a J-domain usually found in co-chaperones. HopI1 localizes to the chloroplast using a non-cleavable transit peptide and the protein targets Hsp70, resulting in modification of thylakoid structures and suppression of SA accumulation [72,73]. Expression of HopI1 in transgenic *A. thaliana* plants expressing high SA levels resulted in a 60% decrease in the level of SA-inducible *PR-1* (Pathogenesis related-1) gene transcript, and around 50% lower free and total levels of SA [72].

HopNI is another well-studied *P. syringae* effector protein that is targeted to the chloroplast using a non-cleavable transit peptide [74]. HopN1 codes for a cysteine protease that cleaves PsbQ, an intrinsic protein of photosystem II in tomato cells, thereby diminishing the photolysis of water [74]. In addition, HopN1 was previously found to suppress cell death associated with HR [75]. It was further demonstrated that HopN1 lacking the catalytic activity of the cysteine protease was unable to inhibit ROS production compared with the wild-type protein [74].

Two other *P. syringae* effectors, AvrRps4 and HopK1, target the chloroplast via a proposed cleavable transit peptide [68]. Specifically, AvrRps4 and HopK1 share sequence similarity in an N-terminal region that may represent the chloroplast transit peptide. AvrRps4 triggers RPS4-dependent immunity in *Arabidopsis,* and transgenic expression of AvrRps4 in *rps4* plants resulted in enhanced growth of *Pst* DC3000 and suppression of PTI [76]. Similar to AvrRps4, HopK1 has been shown to contribute to *Pst* DC3000 virulence. HopK1 activity was also shown to reduce ROS production and callose deposits, indicating that the effector can suppress PTI [68]. 

Although HopI1, HopN1, AvrRps4 and HopK1 are the best-characterized chloroplast-targeted effectors from *P. syringae*, other putative chloroplast-targeted effectors have been identified, including HopBB1 and HopM1 [discussed in [5]. HopBB1 interacts with nuclear and chloroplast proteins and may have more than one intracellular location. HopBB1 also interacts with proteins involved in JA signaling, and with the PTF1 protein that regulates photosynthesis. HopM1 interacts with chloroplast proteins, but some uncertainty remains about its subcellular location [5]. Importantly, HopM1 interacts with MIN7 and MIN10, proteins with known roles in plant immunity.

Chloroplast-targeted effectors are also produced by other bacterial species, including the economically important pathogen *Ralstonia solanacearum*, the causative agent of bacterial wilt diseases in potato, tomato and banana [77]. Similar to *P. syringae*, *R. solanacearum* uses a type three secretion system to facilitate the delivery of more than 70 effector proteins called Rips (Ralstonia injected proteins) into plant cells [69]. One of the best-studied chloroplast-targeted effectors in *R. solanacearum* is RipAL, a protein with a putative lipase domain. RipAL presumably targets chloroplast lipids and is thought to induce JA production and consequently suppress SA-mediated defense responses (PTI) in *N. benthamiana* [69]. Another *R. solanacearum* effector, RipG, contains an F-box domain and is thought to be part of an SCF-type E3 ubiquitin ligase complex, controlling specific protein ubiquitination [78]. The RipG effector family contains seven members that interact with chloroplast proteins, suggesting that these proteins could be targeted for ubiquitination and proteasomal degradation [78]. A chloroplast-targeted effector Las5315 has also been characterized in *Candidatus* Liberibacter asiaticus [79]. This bacterium causes the Huanglongbing (citrus greening) disease of citrus crops typified by chlorosis and starch accumulation. Las5315 acts to upregulate the expression of enzymes involved in starch production and to downregulate functions for starch degradation [79]. Finally, we note that the WtsE effector of *Pantoea stewartii*, a wilt and leaf blight pathogen of maize, has a major impact on chloroplast-associated functions, including secondary metabolism and photosynthesis, although it is not clear that the effector localizes to chloroplasts [80].

### 4.2. Fungal Effectors Targeting Chloroplast Functions

Perhaps the clearest example of a chloroplast-targeted fungal effector comes from the study of *Pyrenophora tritici-repentis*, a fungal pathogen that produces the host-selective toxins ToxA and ToxB to support a necrotrophic attack on wheat [81,82,83]. Plant sensitivity to ToxA is governed by the *Tsn1* locus that encodes an NBS-LRR type R protein, and the toxin is thought to enter host cells by endocytosis and to act in chloroplasts. ToxA induces cell death in a light-dependent manner and provokes ROS accumulation in chloroplasts resulting in a reduction in the levels of PSI and PSII protein complexes. ToxA interacts with the chloroplast protein ToxA Binding Protein 1 (ToxABP1) in wheat, and a homolog designated Thylakoid formation 1 (Thf1) in *A. thaliana*. Thf1 is proposed to play a role in PSII biogenesis or degradation. Interestingly, the severity of tissue necrosis provoked by ToxA can be blocked by preventing the accumulation of ROS or by silencing ToxABP1 in wheat.

Some effectors from rust fungi show sequence signatures with similarity to host transit peptides for translocation into chloroplasts. Examples of these effectors are found in *Melampsora larici-populina*, the poplar leaf rust fungus that causes annual epidemics and severe damage to poplar plantations (especially in Northern Europe) [84,85,86,87]. *M. larici-populina* has been used as a model rust, and its genome was one of the first rust genomes to be sequenced, with 16,399 predicted genes, including genes for candidate secreted proteins. Candidate effectors with a predicted chloroplast transit peptide were recently identified in *M. larici-populina* and designated chloroplast-target proteins (CTP1, CTP2 and CTP3) [88]. Subsequent work revealed that the N-terminal transit peptide, which is cleaved in planta, is sufficient for CTP1, CTP2 and CTP3 accumulation in chloroplasts [89]. Although the impact of the proteins on chloroplast function is just starting to be examined [90], these studies show that fungi have evolved strategies to direct their effectors to the host’s chloroplasts by mimicking the host’s sorting signals. 

Compared with the information on bacterial effectors, there is a paucity of documented localization of fungal effectors in chloroplasts. For fungi, the rarity of chloroplast-targeted effectors is illustrated by a recent review that presented a non-exhaustive list of well-characterized biotrophic and hemibiotrophic fungal effectors [91]. Surprisingly, chloroplast-targeted effectors have not been readily identified in some of the best-characterized fungal pathogens, such as the hemibiotrophic fungus *M. oryzae* [92] or the biotrophic gall-inducing fungus *U. maydis*. For the latter, classic disease symptoms, such as a source-to-sink tissue transition during colonization and the loss of chlorophyll in infected maize tissue, suggest that *U. maydis* produce effectors to target the chloroplast, although these effectors have yet to be discovered. We should note that a recent study identified four RXLR effectors from the oomycete pathogen *Plasmopara viticola* that localize to the chloroplast, although a functional impact remains to be demonstrated [12].

One emerging platform to predict fungal effector proteins, especially when combined with in planta expression data, is EffectorP (http://effectorp.csiro.au/) [93]. Compared to other effector prediction platforms, EffectorP is focused entirely on fungal phytopathogens. Notably, computational methods exist for predicting the subcellular localization of plant proteins, but they perform poorly for effector proteins because the bioinformatic recognition of potential plastid localization signals is confounded by the presence of pro-domains of varying length between the N-terminal signal peptides and translocation signals. The LOCALIZER platform (http://localizer.csiro.au/) was recently developed for predicting effector localization in plants [94]. This approach predicted the chloroplast location of the ToxA protein of *P. tritici-repentis* (discussed above), and the prediction was experimentally confirmed. Additionally, LOCALIZER was employed to survey the secreted proteins of several fungal phytopathogens, and this analysis revealed that rust pathogens have an enrichment of candidate chloroplast effectors compared with other fungi. Subsequent localization experiments identified chloroplast locations for two effectors from *Puccinia graminis* f. sp. *tritici*. Both EffectorP and LOCALIZER have become valuable tools for prioritizing fungal effector candidates for functional investigations. For instance, these two platforms, when used in unison, could provide preliminary predictions for localization of fungal chloroplast-targeted candidate effectors. While most effectors until now have been discovered by genetic and biochemical strategies, computational predictions of effector proteins may provide a faster approach to identify novel candidate effectors. 

## 5. Conclusions and Future Work

Plastids and, more specifically, chloroplast-located processes are potentially important targets of microbial effectors. The relevance of plastids and chloroplasts for plant metabolism and immunity is clear and experimentally validated plastid-targeting fungal and oomycete effectors are emerging. Diseases caused by fungal pathogens, or interactions with beneficial fungi, impact specific chloroplast activities for photosynthesis, chlorophyll biosynthesis and stability, organelle position and the formation of ROS. Although there are well-characterized interactions between viral or bacterial proteins and chloroplast proteins, little is known about the influence of specific fungal effectors that target plastids. In addition, the impact of effectors from fungi and other pathogens on retrograde signaling is under-explored and requires additional investigation. Whereas bioinformatic prediction approaches based on the known plastid-translocation signals can be the basis for targeted experimental searches for plastid-localized effectors, non-classical organelle targeting of effectors should not be excluded. Therefore, non-targeted systematic effector localization screens and biochemical approaches to co-purify effectors with organelle-preparations followed by mass spectrometry might lead to exciting discoveries. 

Although fungal effectors are now being identified in chloroplasts, questions remain about the biochemical state of these effectors and how they reach their subcellular destination. The translocation of proteins from the plant cytosol to plastids occurs for polypeptides in an unfolded state and is a GTP- and ATP-driven process. Plant precursor proteins are kept in an unfolded, translocation-competent state by chaperones of the hsp70 family. How fungal plastid-effectors first get secreted from the fungal cells and then passage through the biotrophic interface safely, translocate into the host cytoplasm, and finally get translocated into plastids is enigmatic and awaits future research. Overall, effector research will deepen our understanding of the relevance of plastids in biotic interactions and highlight potential central regulatory hubs located in plastids.
